# Transgenerational Epigenetic Effect of Cryopreservation of F0 Rooster Sperm (*Gallus gallus domesticus*) on microRNA-Regulation and Histological Parameters of the Reproductive System of F1 Offspring

**DOI:** 10.3390/ani16111723

**Published:** 2026-06-04

**Authors:** Anastasiya Ivershina, Yuliya Silyukova, Elena Fedorova, Elena Chugunova, Irina Mirzakaeva, Anna Modina, Olga Stanishevskaya

**Affiliations:** Russian Research Institute of Farm Animal Genetics and Breeding—Branch of the L.K. Ernst Federal Research Center for Animal Husbandry (RRIFAGB), Tyarlevo, Moskovskoe Shosse, 55a, 196625 St. Petersburg, Russiafarra.kild@yandex.ru (I.M.);

**Keywords:** epigenetic transgenerational effect, microRNA, *DMRT1*, *TGFB2*, cryopreservation, sperm, gonads, *Gallus gallus domesticus*, histomorphometry

## Abstract

Sperm cryopreservation is an integral part of gene pool conservation programs for local poultry breeds. It is known that cryostress can cause significant changes in the expression profiles of microRNAs and their target genes—key players in spermatogenesis—in *Gallus gallus domesticus*. However, the transmission of these changes across generations remains poorly understood. The aim of this study was to evaluate the transgenerational epigenetic effects of rooster sperm cryopreservation on molecular genetics and histological parameters in the gonads of offspring (F1) during the embryonic (10 days) and postnatal (1 day) periods. The analysis included a comprehensive histomorphometric analysis of the gonads and a quantitative assessment of the expression of microRNAs (gga-miR-6701-3p, gga-miR-301a-5p) and their target genes (*DMRT1*, *TGFB2*), using qRT-PCR. Histological analysis of the gonads of 10-day-old embryos revealed early morphological abnormalities in the F1 (n = 10) offspring obtained from frozen–thawed semen (experimental group). It was found that day-old F1 chicks (n = 17) obtained from frozen semen had testes with a significantly reduced number of seminiferous tubules (−36%, *p* < 0.05) with an increased diameter (+22%, *p* < 0.05) and an increased number of undifferentiated gonocytes (+53%, *p* < 0.001) compared to chicks obtained from native semen (control group, n = 20). A decrease in the expression of *DMRT1* and *TGFB2* in the gonads of embryos (−48% and −29%, respectively, *p* < 0.05) and day-old chicks (−12% and −43%, *p* < 0.001 for *TGFB2*) was found, accompanied by an inversion of microRNA dynamics: miR-6701-3p was decreased and miR-301a-5p was increased. The obtained data provide important evidence of transgenerational effects in birds and contribute to the search for solutions to problems associated with maintaining sperm quality after cryopreservation, and indicate that cryopreservation does not simply reduce the level of molecular activity, but disrupts the ontogenetic regulatory program embedded in the genome.

## Simple Summary

Sperm cryopreservation is an integral part of gene pool conservation programs for local poultry breeds. Cryostress is known to cause significant changes in the expression profiles of microRNAs and their target genes—key players in spermatogenesis—in *Gallus gallus domesticus*. The transgenerational epigenetic effects of rooster sperm cryopreservation on molecular genetics and histological parameters in the gonads of offspring (F1) were studied during the embryonic (10 days) and postnatal (1 day) periods. Early morphological abnormalities were identified in F1 offspring obtained from cryopreserved semen (experimental group). One-day-old chicks obtained from frozen semen had testes with a significantly reduced number of seminiferous tubules with an increased diameter and an increased number of undifferentiated gonads compared to chicks obtained from native semen. A decrease in *DMRT1* and *TGFB2* expression was detected in the gonads of embryos and day-old chicks, accompanied by an inversion of microRNA dynamics: miR-6701-3p was downregulated, while miR-301a-5p was upregulated. These findings provide important evidence of transgenerational effects in birds and contribute to the search for solutions to problems associated with maintaining sperm quality after cryopreservation in poultry farming.

## 1. Introduction

In recent years, the molecular mechanisms underlying the decline in sperm quality after cryopreservation have become the focus of intense research. While previously, the primary focus was on optimizing cryoprotective media and freezing protocols [1,2], the current stage of development in reproductive biotechnology is characterized by a shift toward studying the intergenerational consequences of prezygotic stress on subsequent generations. Our previous studies [3] demonstrated for the first time that cryopreservation of *Gallus gallus domesticus* rooster semen induces significant changes in the expression of microRNAs and their target genes in the sperm of the parental generation (F0). In particular, an increase in the expression level of gga-miR-301a-5p was detected, while the expression of its target gene *TGFB2* was decreased (*p* < 0.05), as well as a significant suppression of the expression of gga-miR-6701-3p (*p* < 0.001) in cryopreserved sperm. These data indicate that cryopreservation disrupts the coordinated regulation of gene networks that control spermatogenesis and the functional activity of germ cells. Direct experimental evidence has been published in the scientific literature demonstrating that spermatozoa are capable of carrying not only the haploid genome, but also epigenetic information in the form of chromatin modifications, DNA methylation, and the profile of small non-coding RNAs [4,5,6]. Sperm microRNAs are delivered to the oocyte during fertilization and are necessary for the first division of the zygote [4], and their absence leads to disturbances in preimplantation development [5]. For example, it has been demonstrated in mammals that microRNAs expressed in sperm are delivered to the oocyte during fertilization and participate in the regulation of early embryogenesis [7,8]. It is also known that exposure to extremely low temperatures alters the activity of microRNAs in sperm, which affects early embryonic development [9,10]. However, in farming, this aspect remains virtually unexplored. The peculiarities of avian reproductive biology (meroblastic cleavage, early sex determination by the ZZ/ZW genotype, specific chromatin organization in sperm) make direct extrapolation of data from mammals incorrect. In particular, the *DMRT1* gene, localized on the Z chromosome, is a key regulator of male determination in birds and exhibits dose-dependent expression in the early stages of embryogenesis [11,12]. Another gene under study, *TGFB2*, activates the SMAD signaling pathway and is involved in Sertoli cell differentiation and seminiferous tubule formation [13,14]. We hypothesize that the identified abnormalities in the expression profiles of microRNAs and their target genes, mediated by exposure to ultra-low temperatures, may have long-term consequences for the reproductive health of offspring.

The aim of this study was to conduct a comparative analysis of the expression of gga-miR-301a-5p and gga-miR-6701-3p microRNAs, as well as their target genes *TGFB2* and *DMRT1*, in the reproductive tissues of F1 offspring (in the gonads of 10-day-old embryos and in the testes of day-old chicks) obtained from insemination of laying hens with native and cryopreserved semen of F0 roosters, to assess the intergenerational effects of prezygotic stress at the molecular level.

## 2. Materials and Methods

### 2.1. Animal Husbandry

The study was conducted in 2025 using Russian Snow White egg-laying roosters. F0 roosters (n = 10) were selected to produce F1 offspring using native (control group) and frozen–thawed (experimental group) semen at 24–28 weeks of age based on native semen quality assessment (ejaculate volume was 0.3–0.9 mL, semen concentration was at least 1.5 billion/mL, and total motility was at least 70%). The roosters were maintained under identical housing and feeding conditions in individual cages. From the age of sexual maturity (at least 24 weeks), the roosters were accustomed to abdominal massage and used for semen collection twice a week. Semen samples were collected every 3 days in the morning and immediately delivered to the laboratory (6 consecutive collections were carried out, every second semen sample was cryopreserved).

### 2.2. Semen Cryopreservation

The resulting ejaculate was diluted using the Leningradskaya cryoprotective medium (LCM) [15] at a 1:1 ratio. LCM composition (per 100 mL of distilled water): monosodium glutamate (1.92 g), fructose (0.8 g), potassium acetate (0.5 g), polyvinylpyrrolidone (0.3 g), and protamine sulfate (0.032 g). Samples were equilibrated from 18 °C to 5 °C for 40 min. After cooling, dimethylacetamide (DMA, SigmaAldrich, St. Louis, MO, USA) was added to each sample at a final concentration of 6%. After adding DMA, the samples were exposed to 5 °C for 1 min. Freezing was performed in pellets by directly dropping the sperm into liquid nitrogen. The position of the pipette with semen was controlled using a digital thermocouple with a sensor (THERM 2420, AHLBORN, Holzkirchen, Germany), and in the area where the pipette was located, it ranged (°C) from −15 to −20 °C. The average pipetting speed was ~1.4 pellets per second. Thawing of the pellets was performed on a heated metal plate at a temperature of 60 °C (slot defroster, developed by RRIFAGB 1989). Freezing of every second sample was carried out for 1 month.

### 2.3. Semen Quality Assessment

The quality of native and thawed semen samples from F0 experimental roosters was assessed using the following parameters:

– Concentration (billion/mL, Accuread Photometer, IMV Technologies, Bellshill, UK; three replicates for each individual);
– Total motility (TM) and progressive motility (PM) (%), CASA computerized semen analysis system (Motic BA410E, Motic China Group Co., Ltd. Xiamen, Fujian, China; negative contrast, ×100; BASLER acA1300 digital image input system) and ArgusSoft-Poultry-1 software (ArgusSoft LLC, St.-Petersburg, Russia, 2021).

Quality assessment of native and frozen–thawed semen (F0) by flow cytometry methods:

– The content of live cells and cells in apoptotic state (%) was determined using the V-AF488 flow cytometry kit (Lumiprobe RUS LLC, Moscow, Russia). The cells were washed with cold PBS (pH 7.4) and annexin binding buffer, then resuspended in cold annexin binding buffer. Afterwards, 100 μL of cell suspension were collected (from 1.5 × 10^5^ to 1.5 × 10^6^ cells/mL) in 1.5 mL microcentrifuge tubes. Then, 2–5 μL of annexin V-AF488 solution were added to each tube and incubated for 10–15 min at room temperature, protected from light. Without preliminary washing, 400 μL of annexin binding buffer were added to each tube and 5 μL of propidium iodide were added. The contents of the tube were gently mixed and incubated for 5 min at room temperature, protected from light. Stained cells were stored at 2–8 °C, protected from light, for no more than 2 h. The number of events assessed on a Cytoflex flow cytometer (Beckman Coulter, Inc., Brea, CA, USA) was 1000–1500 for each semen sample. The integrity of mitochondria and their membrane potential in living cells was assessed using the mitochondrial marker Mito TMRE (tetramethylrhodamine, ethyl ester; OOO Lumiprobe RUS, Moscow, Russia) according to the proposed protocol on a Cytoflex flow cytometer (Beckman Coulter, Inc., Brea, CA, USA).

### 2.4. Obtaining F1 Offspring

To obtain F1 offspring, 10 groups of virgin hens (1♂: 5♀) were formed. The hens were kept in individual cages under the conditions adopted by the Collective Use Center “Genetic Collection of Rare and Endangered Chicken Breeds” (https://vniigen.ru/ckp-geneticheskaya-kollekciya-redkix-i-ischezayushhix-porod-kur/ (accessed on 19 March 2026)). The hens were inseminated intravaginally with thawed semen for two weeks according to the following schedule: a single dose of 0.04–0.07 mL of thawed semen (the insemination dose contained at least 70–80 million motile spermatozoa) on two consecutive days, and then every three days thereafter. A total of five inseminations were performed. Then, a three-week break was taken, the absence of fertilized eggs was monitored for two days, and then insemination with native semen was performed according to the same insemination schedule. The eggs were incubated in a RAMEEL 400C laboratory incubator (RAMEEL, Ryazan, Russia), under the standard incubation mode for chicken eggs (temperature 37.5 °C, humidity 55.0%).

F1 offspring obtained from native semen constituted the “F1 control group”; F1 offspring obtained from frozen–thawed semen constituted the “F1 experimental group.”

### 2.5. Collection of Biological Samples of Gonads from 10-Day-Old Embryos and 1-Day-Old Chicks

For molecular genetics and histological analysis, testes from 10-day-old embryos and 1-day-old chicks obtained from both native and frozen–thawed semen were collected. Gonads were obtained by removal from the abdominal cavity. Euthanasia of 10-day-old embryos (from native semen n = 15; from frozen–thawed semen n = 10) and 1-day-old chicks (from native semen n = 20; from frozen–thawed semen n = 17) was performed by decapitation (the animal study protocol was approved by the Ethics Committee of the Russian Research Institute of Genetics and Breeding of Farm Animals—a branch of the L.K. Ernst Federal Research Center for Animal Husbandry (RRIFAGB), protocol No. 1, dated 13 January 2025).

### 2.6. Preparation and Analysis of Histological Specimens from 10-Day-Old Embryos and 1-Day-Old Chicks (F1)

The specimens were fixed in Bouin’s solution for 24 h. The fixed specimens were embedded in paraffin, serially sectioned at 4 μm thickness on an RMD-3000 microtome (MT Point, Russia), stained with hematoxylin/eosin, and analyzed microscopically (magnification: 100×, 400×, and 1000×, Bioskop-4 microscope, LOMO, St.-Petersburg, Russia).

In histological sections of gonads from 10 day-old embryos, gonad length (µm), gonad width (µm), and the number of gonocytes (pcs.) were assessed in three consecutive fields of view over an area of 10,000 µm^2^.

In histological sections of testes from day-old chicks, testicle length (µm), testicle width (µm), number of seminiferous tubules in cross-section (pcs.), diameter of seminiferous tubules in cross-section (µm), height of spermatogenic epithelium of testis (µm), and number of spermatogonia in the cross-section of seminiferous tubule (pcs.) were assessed in three consecutive fields of view.

### 2.7. RNA Isolation

To isolate sufficient quantities of microRNA from the gonadal tissues of 10-day-old embryos, gonads from full siblings (n = 5) were used to isolate total RNA from a single sample (n = 3 samples from native semen; n = 2 samples from frozen–thawed semen). Total RNA was isolated from testicular and embryonic gonad tissues (F1) using the Lira Karib reagent kit (Biolabmix LLC, Moscow, Russia) according to the manufacturer’s instructions. The isolation steps included homogenization of the samples using ceramic beads on a Precellys 24 homogenizer (Bertin Technologies, Montigny-le-Bretonneux, France), purification from DNA and other contaminants, column washing with WB buffer, and RNA elution with EB buffer. The quality and concentration of the obtained RNA were assessed spectrophotometrically using a NanoDrop 2000 instrument (Thermo Fisher Scientific, Waltham, MA, USA) based on the ratio of optical densities at wavelengths of 260 and 280 nm (purity ratio A260/A280 = 1.8–2.1). Only samples that met the following quality criteria were included in the analysis: no signs of degradation (visualization of 28S/18S rRNA) and a purity ratio in the range of 1.8–2.1.

### 2.8. Relative Gene Expression Analysis

cDNA synthesis and quantitative expression analysis were performed separately for microRNAs and their target genes, taking into account their structural features. For microRNAs (gga-miR-6701-3p, gga-miR-301a-5p), the stem-loop reverse transcription method was used with specific primers (Table 1). Amplification and detection were performed using hydrolyzable TaqMan probes from commercial TaqMan™ MicroRNA Assays (Thermo Fisher Scientific, Waltham, MA, USA). Small nuclear RNA U6 (RNU6B) was used as an endogenous control. For microRNAs of target genes (*DMRT1*, *TGFB2*), cDNA synthesis was performed using oligo (dT) primers and reverse transcriptase from the M-MuLV–RH kit (Biolabmix, Moscow, Russia). Quantitative PCR was performed using the SYBR Green intercalating dye (BioMaster RT-PCR SYBR Blue (2×), Biolabmix LLC, Moscow, Russia). The housekeeping gene *GAPDH*, whose stable expression in avian reproductive tissues had been confirmed in preliminary studies, was used as an endogenous control [16]. Primers for microRNAs were designed using the miRNA Primer Design Tool (http://www.srnaprimerdb.com/). Primer sequences for the *DMRT1*, *TGFB2*, and *GAPDH* genes were selected using Primer BLAST (https://www.ncbi.nlm.nih.gov/).

All amplification reactions were performed on a QuantStudio™ 5 Real Time PCR System (Thermo Fisher Scientific, Waltham, MA, USA) in triplicate for each sample. Amplification conditions included pre-denaturation at 95 °C for 10 min, followed by 40 cycles (denaturation at 95 °C for 15 s, annealing–elongation at 60 °C for 1 min). The specificity of amplification using SYBR Green was monitored by melting curve analysis and confirmed by electrophoresis in a 2% agarose gel.

Relative expression levels were calculated using the delta–delta cycle threshold (2^−ΔΔCt^) method with normalization to the corresponding endogenous controls. For microRNAs, normalization was performed to the mean U6 value; for gene mRNAs, to *GAPDH*.

### 2.9. Statistical Analysis

Group comparisons between native and cryopreserved sperm groups were assessed using the Mann–Whitney U test for day-old chicks and Student’s *t*-test for embryos. Differences were considered statistically significant at *p* ≤ 0.05 (adjusted for multiple comparisons). All statistical analyses were performed using GraphPad Prism 12.0 (GraphPad Software, San Diego, CA, USA) and Python 3.x (scipy and statsmodels packages). Data visualization was conducted in Python using matplotlib and seaborn libraries.

## 3. Results

### 3.1. Semen Quality Assessment of Roosters (F0)

Quality indicators for native and frozen–thawed semen samples are presented in Table 2.

Overall sperm motility decreased by 33.7% (*p* < 0.001), while progressive motility decreased by 44.7% (*p* < 0.001). The smallest decrease was observed in viability, at 23.4%.

Flow cytometry using double staining (Annexin V-FITC/propidium iodide) confirmed these results. The proportion of intact cells (Annexin V^−^/PI^−^) decreased by 30.03% (*p* < 0.001). In addition, a significant increase in the proportion of early apoptotic spermatozoa (Annexin V^+^/PI^−^) by 2.64% (*p* < 0.001) was observed (Table 3).

Mitochondrial activity of spermatozoa, assessed by the accumulation of the voltage-dependent dye TMRE, also significantly decreased after cryopreservation by 23.26% (*p* < 0.001). This indicates damage to mitochondrial membranes and a decrease in sperm energy potential.

The decrease in sperm functional parameters after cryopreservation directly affected reproductive performance. With insemination using native semen, egg fertilization rates were over 90%, while with frozen–thawed semen, this rate ranged from 40.6% to 63.2%.

### 3.2. Histological Structure of Embryonic Gonads and Testes of 1-Day-Old Chicks

#### 3.2.1. Histological Structure of the Gonads of 10-Day-Old Embryos

**Figure 1:**
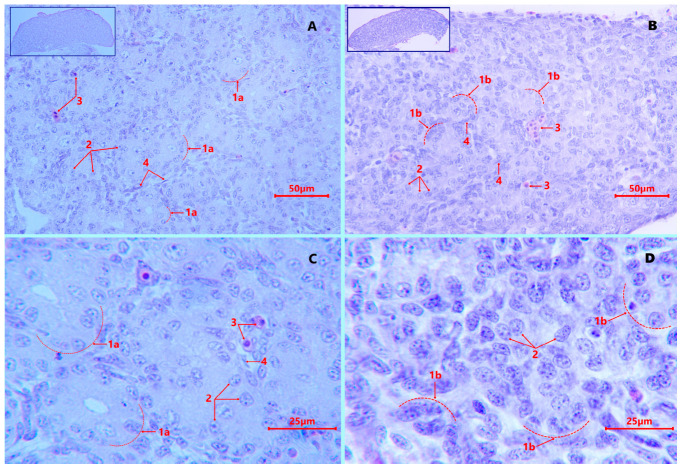
Fragments of histological preparations of gonads: (**A**) 10-day-old F1 embryos (control group; magnification ×400); (**B**) 10-day-old F1 embryos (experimental group; magnification ×400); (**C**) 10-day-old F1 embryos (control group; magnification ×1000 oil); (**D**) 10-day-old F1 embryos (experimental group; magnification ×1000 oil). 1a, 1b—medullary cords; 1a—densely packed medullary cords; 1b—disorganization of medullary cords; 2—gonocytes; 3—red blood cells in the vascular lumen; 4—embryonic myoid cells.

Histological analysis of the gonads of 10-day-old embryos revealed early morphological abnormalities in the F1 offspring obtained from cryopreserved semen. In the control group, the gonads had a structure typical for this stage of ontogenesis: a symmetrical arrangement of medullary cords, densely packed gonads, Sertoli pre-cells, and seminiferous tubule rudiments (Figure 1A). In the experimental group, disorganization of the medullary cords was observed—an uneven distribution of cell clusters, an increase in interstitial space, and a decrease in gonad cell density.

Histomorphometric analysis of the gonads of 10-day F1 embryos did not reveal significant statistical differences between the groups, but there was a tendency towards increased variability in the experimental group (CV = 18.8% for gonad length) (Table 4).

#### 3.2.2. Histological Structure of the Testes of 1-Day-Old Chicks

**Figure 2:**
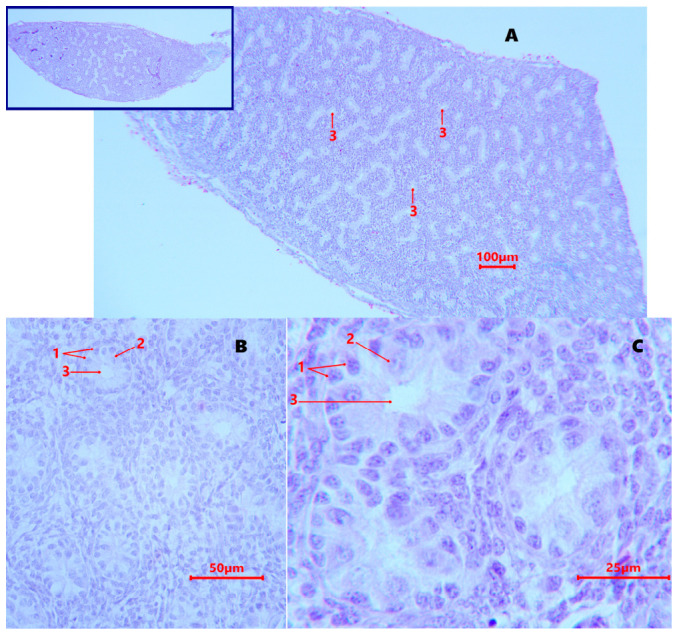
Fragment of a histological preparation of a testis (F1-control group—1-day-old chick obtained from native sperm): (**A**) long section of the testis (magnification ×200); (**B**) transverse section of the seminiferous tubule (magnification ×400); (**C**) transverse section of the seminiferous tubule (magnification ×1000 oil). 1—gonocytes; 2—Sertoli cell precursors; 3—lumen of the canal.

**Figure 3:**
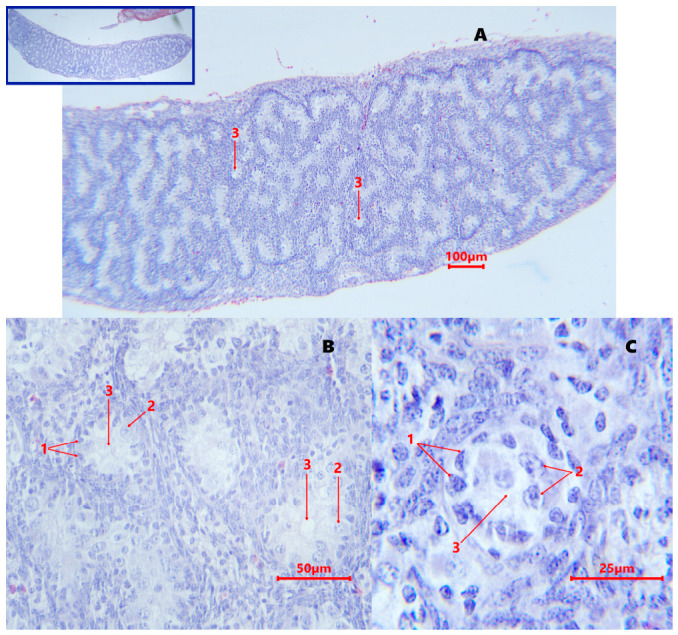
Fragment of a histological preparation of a testis (F1 experimental group—1-day-old chick obtained from frozen–thawed semen): (**A**) long section of the testis (magnification ×200); (**B**) transverse section of the seminiferous tubule (magnification ×400); (**C**) transverse section of the seminiferous tubule (magnification ×1000 oil). 1—gonocytes; 2—Sertoli cell precursors; 3—lumen of the canal.

Histological analysis of the testes of day-old F1 chicks revealed significant differences in testicular tissue architecture between the control (Figure 2A) and experimental (Figure 3A) groups. The testes of 1-day-old chicks in the control group were characterized by well-defined seminiferous tubule structure with a uniform distribution of spermatogenic epithelial cells (Figure 2B).

In the experimental group, disorganization of the structure was observed: the structure of the tubules was not clearly expressed, with an uneven distribution of gonocytes (Figure 3B).

Despite some chaotic organization of the seminiferous tubule epithelium in the testis samples from the experimental group (Figure 3C), the spermatogonia density in the lumen of the seminiferous tubule cross-section was visually higher than in the control group (Figure 2C).

The results (Table 5) revealed morphological differences between the experimental and control groups: the experimental group had a significantly (*p* < 0.05) lower number of seminiferous tubules in the testis cross-sections compared to the control group, while the diameter of the seminiferous tubules was significantly larger by 18.4% (*p* < 0.05). This is presumably due to decreased cord formation in the gonads during embryonic development, which subsequently affected the formation of the seminiferous tubules. In addition, the number of gonocytes calculated per cross-section of one seminiferous tubule (standardized field of view at ×1000 oil magnification) was significantly higher in the experimental group (*p* < 0.001), reflecting the increased density of cell packing in the dilated tubules.

### 3.3. Analysis of Relative Gene Expression in the Gonads and Testes of F1 Offspring

**Figure 4:**
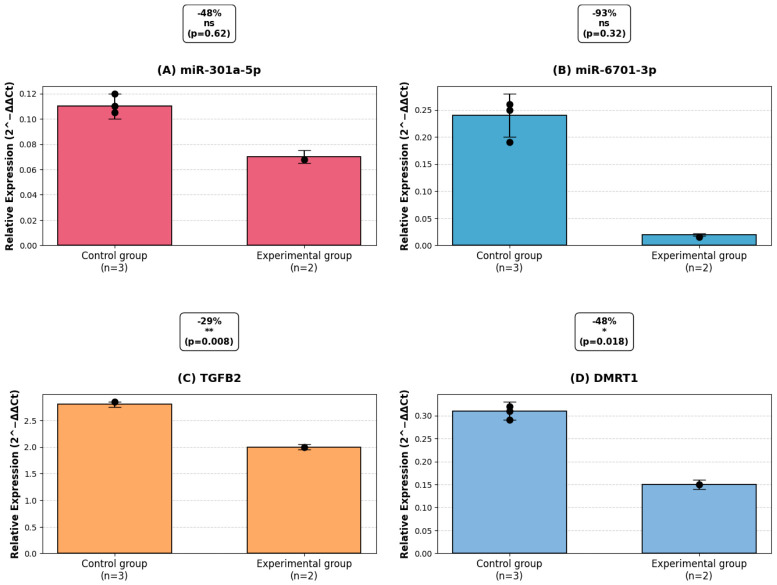
microRNA and target gene expression in F1 10-day embryonic gonads (control group vs. experimental group). Relative expression (2^−ΔΔCt^) of (**A**) miR-301a-5p, (**B**) miR-6701-3p, (**C**) *TGFB2*, and (**D**) *DMRT1* in gonads of 10-day embryos obtained from control group (n = 3) or experimental group (n = 2) sperm. Bar graphs show mean ± SEM; individual data points are overlaid. Statistical significance determined by Student’s *t*-test. * *p* < 0.05, ** *p* < 0.01, ns = not significant. Note: Small sample size (n = 2–3) limits statistical power for miRNA comparisons.

Quantitative assessment of the expression of key male differentiation genes in the gonads of 10-day-old embryos revealed a significant suppression of their transcriptional activity in offspring obtained from frozen–thawed semen (experimental group). Thus, the expression level of the *TGFB2* gene, involved in the regulation of testicular development and differentiation of somatic gonadal cells, was reduced by 29% in the experimental group compared to the control (2^−ΔΔCt^= 1.996 ± 0.062 vs. 2.809 ± 0.069; t = 6.32; *p* = 0.008) (Figure 4A,C).

The decrease in the expression of the *DMRT1* gene, a key regulator of male sex determination in birds [17], turned out to be more pronounced: its level in the gonads of embryos in the experimental group was only 52% of the control values (2^−ΔΔCt^ = 0.153 ± 0.008 versus 0.291 ± 0.017; t = 4.68; *p* = 0.018, Figure 4B,D).

The microRNA profile in embryonic gonads was characterized by a decrease in both studied molecules, although statistical significance was not achieved due to the limited sample size. The expression of gga-miR-6701-3p was reduced by 93% in the experimental group (2^−ΔΔCt^ = 0.017 ± 0.001 vs. 0.254 ± 0.127; U = 1; *p* = 0.32), while gga-miR-301a-5p showed a moderate decrease of 48% (2^−ΔΔCt^ = 0.067 ± 0.047 vs. 0.128 ± 0.062; U = 2; *p* = 0.62). Although there were no significant differences for microRNAs, the observed direction of changes (decrease in both molecules with simultaneous suppression of their target genes) suggests disruption of classical regulatory interactions. In particular, the reduction in gga-miR-6701-3p with simultaneous suppression of *DMRT1* contradicts the expected upregulation and likely indicates the dominance of epigenetic mechanisms of suppression (e.g., hypermethylation of the *DMRT1* promoter [18,19]) induced by cryostress in the parental generation.

**Figure 5:**
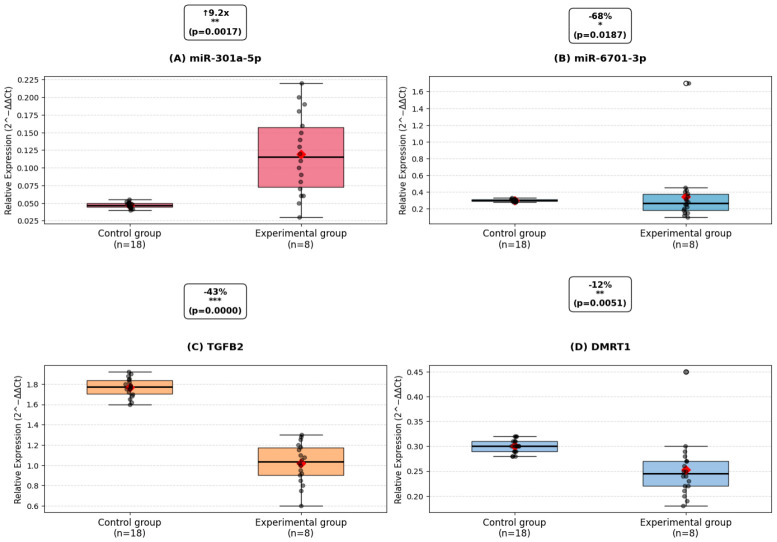
microRNA and target gene expression in F1 1-day-old chick testes (native vs. cryopreserved sperm). Relative expression (2^–ΔΔCt^ of (**A**) miR-301a-5p, (**B**) miR-6701-3p, (**C**) *TGFB2*, and (**D**) *DMRT1* in testes of day-old chicks obtained from native (n = 18) or cryopreserved (n = 8) sperm. Box plots show median, quartiles, and range; individual data points are overlaid. Red diamonds indicate group means. Statistical significance determined by Mann–Whitney U test. *** *p* < 0.002, ** *p* < 0.01, * *p* < 0.05.

By the age of one day, a change in the direction of regulation of one of the key microRNAs was observed in the testes of the offspring, which reflects the dynamic nature of the transgenerational effects of cryopreservation. Expression of gga-miR-6701-3p remained significantly reduced in the experimental group by 68% (2^−ΔΔCt^= 0.110 ± 0.022 vs. 0.347 ± 0.034; U = 68; *p* < 0.0001; n = 8 and 18, respectively) (Figure 5A,C). At the same time, expression of gga-miR-301a-5p demonstrated the opposite dynamics—its level in the experimental group was increased by 9.2 times compared to the control (2^−ΔΔCt^ = 0.395 ± 0.181 vs. 0.043 ± 0.006; U = 42; *p* = 0.0002), which corresponds to the classical mechanism of negative regulation of its target gene *TGFB2* [13]. The expression of the *TGFB2* gene was significantly reduced by 43% in the testes of day-old chicks obtained from thawed semen (2^−ΔΔCt^ = 1.037 ± 0.070 versus 1.809 ± 0.079; U = 38; *p* < 0.0001), which directly correlated with the identified histological abnormalities: a decrease in the number of seminiferous tubules by 36% (*p* < 0.05), an increase in their diameter by 22% (*p* < 0.05) and an increase in the number of undifferentiated gonocytes by 53% (*p* < 0.001). *DMRT1* gene expression at 1 day of age showed only a 12% decrease trend (2^−ΔΔCt^ = 0.270 ± 0.026 vs. 0.306 ± 0.016; *p* = 0.189), indicating partial compensation of the critical sex determination gene in the postnatal period, probably due to the activation of alternative regulatory pathways (e.g., Notch/GDNF) (Figure 5B,D) [20].

### 3.4. Transgenerational Dynamics of MicroRNA and Gene Expression in the F0 → F1 Generation Series

**Figure 6:**
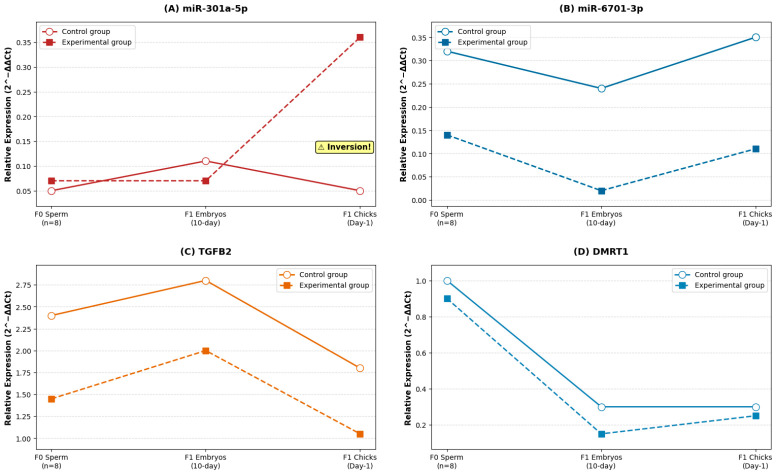
Transgenerational dynamics of microRNA and gene expression: F0 Sperm (n = 8) → F1 Embryos → F1 1-day-old chicks. Relative expression (2^−ΔΔCt^) across developmental stages for (**A**) miR-301a-5p, (**B**) miR-6701-3p, (**C**) *TGFB2*, and (**D**) *DMRT1*. Solid lines with circles represent control group; dashed lines with squares represent experimental group. Note the expression inversion of miR-301a-5p in the experimental group between embryonic and post-hatching stages (yellow warning symbol in (**A**)). This indicates disruption of ontogenetic regulation programs by sperm cryopreservation.

To assess the nature of the transmission of molecular changes from the parental generation (F0) to the offspring (F1), we conducted a comparative analysis of the expression of microRNAs and their target genes at three key stages: sperm of father roosters (F0), gonads of 10-day-old embryonic offspring (F1), and testes of day-old chick offspring (F1) (Figure 6A–D).

#### 3.4.1. miR-301a-5p and TGFB2: Inversion of the Developmental Pattern

The most pronounced transgenerational effect was demonstrated by miR-301a-5p, a microRNA that regulates *TGFB2* gene expression (Figure 6A,C). In the control group, physiological expression dynamics were observed: miR-301a-5p expression levels increased in parental sperm (F0) (0.050 ± 0.008) and in gonadal tissues at the embryonic stage (0.111 ± 0.015), after which they decreased in the testes of day-old chicks (0.043 ± 0.006). *TGFB2* gene expression followed the opposite dynamics, consistent with the canonical mechanism of miRNA-mediated repression. A paradoxical pattern was recorded in the experimental group: miR-301a-5p expression remained low during embryogenesis (0.067 ± 0.012), but significantly increased by the age of one day (0.395 ± 0.181; *p* < 0.001). Accordingly, *TGFB2* expression in the testes of day-old chicks from the experimental group was reduced by 43% compared to the control (1.037 ± 0.070 vs. 1.809 ± 0.079; *p* < 0.0001). Thus, we can conclude that cryopreservation led to the inversion of the ontogenetic regulatory program of the miR-301a-5p → *TGFB2* pair during postnatal development.

#### 3.4.2. miR-6701-3p and DMRT1: Stable Downregulation Across Generations

The expression of miR-6701-3p, a microRNA that regulates the *DMRT1* gene, was consistently downregulated in the experimental group at all stages studied (Figure 6B,D). In parental sperm, the level of this microRNA was reduced by 59% compared to the control (0.142 ± 0.035 vs. 0.347 ± 0.040; *p* < 0.001). This trend was maintained in the gonads of offspring embryos in the experimental and control groups (a decrease of 93%; 0.017 ± 0.003 versus 0.254 ± 0.127; *p* = 0.008, respectively) and in the testes of day-old offspring chicks (a decrease of 68%; 0.110 ± 0.022 (experiment) versus 0.347 ± 0.034 (control); *p* < 0.0001). *DMRT1* gene expression demonstrated a stage-specific response to cryopreservation. The most pronounced differences between the groups were observed at the embryonic stage: in the experimental group, the *DMRT1* gene expression level was reduced by 48% compared to the control (0.153 ± 0.008 versus 0.291 ± 0.017; *p* = 0.018, respectively). By the age of one day, the differences smoothed out and became statistically insignificant (0.270 ± 0.026 versus 0.306 ± 0.016; *p* = 0.189). In the spermatozoa of the parental generation, the differences also did not reach the level of statistical significance (*p* > 0.05). Thus, cryopreservation of rooster sperm led to the formation of multidirectional molecular changes in a series of generations F0 → F1. The miR-6701-3p → *DMRT1* pair is characterized by a stable transmission of the suppression effect from parents to offspring at all the stages of development studied. In contrast, the miR-301a-5p → *TGFB2* pair demonstrated a paradoxical inversion of the expression pattern in the postembryonic period, which may indicate that cryopreservation disrupts the normal developmental program regulating the reproductive system of the offspring.

## 4. Discussion

In our previous study [3], we analyzed the expression profile of the microRNAs gga-miR-6701-3p, gga-miR-301a-5p, and their target genes *DMRT1* and *TGFB2* in native and cryopreserved semen of F0 parent stock. Significant changes in the microRNA profile were detected in the semen of Russian Snow White roosters after cryopreservation. The expression of gga-miR-301a-5p increased by 84% (2^−ΔΔCt^ = 1.84 ± 0.21; *p* < 0.05), which was accompanied by a decrease in the expression of its target gene *TGFB2* by 42% (2^−ΔΔCt^ = 0.58 ± 0.09; *p* < 0.05), confirming the predicted negative regulatory relationship. Moreover, the expression of gga-miR-6701-3p decreased by 59% (2^−ΔΔCt^ = 0.41 ± 0.07; *p* < 0.001), while its target gene *DMRT1* remained relatively stable (a decrease of only 8%, not significant; 2^−ΔΔCt^ = 0.92 ± 0.08; *p* > 0.05). This indicates compensatory mechanisms for maintaining the expression of a critically important sex determination gene.

This study, a logical continuation of previous work, demonstrates for the first time that cryopreservation of rooster semen induces transgenerational changes in the expression levels of microRNAs and their target genes, which are maintained in F1 offspring throughout development—from the embryonic stage to the postnatal period. The data obtained indicate that cryodamage to spermatozoa affects not only direct indicators of semen quality at the cytological level but also molecular programs regulating the development of the reproductive system in the next generation. As expected, cryopreservation resulted in a significant reduction in all assessed functional parameters of rooster semen. Overall sperm motility in the freeze–thaw cycle decreased from 86.4% to 52.7% (*p* < 0.001), progressive motility—from 78.9% to 34.2% (*p* < 0.001), viability—from 91.7 ± 1.2% to 68.3 ± 3.5% (*p* < 0.001). These disorders are associated with many factors: the formation of ice crystals, exposure to oxidative stress, damage to plasma membranes in cells [21,22] and an increase in the number of cells with programmed death (apoptosis), which is associated with the unique structure of reproductive cells of male birds—a thin layer of cytoplasm, a large area of surface membranes, a high content of cholesterol [23,24,25,26] and polyunsaturated fatty acids in membranes [27]. Mitochondrial activity decreased from 76.52% to 53.26% (*p* < 0.001), indicating mitochondrial-dependent sperm apoptosis as a result of low-temperature exposure [28,29]. Damage to the mitochondrial apparatus leads to a decrease in ATP production, which directly affects the motility of germ cells after thawing [30].

Analysis of the expression profiles of microRNAs and their target genes in the reproductive tissues of offspring demonstrated a stable transmission of molecular genetic changes from the parental generation (F0) to the offspring (F1). In frozen–thawed rooster semen, the expression level of miR-301a-5p was increased by 84% (*p* < 0.05), and in the testes of day-old chicks from these roosters, the expression of the same microRNA was 9.2-fold higher than the control (*p* = 0.0002). This pattern is consistent with the concept of epigenetic inheritance through spermatozoa described for mammals [31,32]. Although direct evidence of the transfer of sperm microRNAs into the egg cytoplasm is absent in birds, data on mammals suggest a similar mechanism in chickens [32,33,34,35]. Avian spermatozoa, despite a high degree of chromatin condensation and a minimal amount of cytoplasm [25], contain functional microRNAs that can be introduced into the zygote during fertilization and regulate early expression of embryonic genes.

The most striking result of our study is the inversion of the miR-301a-5p expression pattern between the embryonic and postnatal stages in the experimental group. In the control group, miR-301a-5p expression increased from sperm (0.043 ± 0.008) to embryos (0.128 ± 0.062), after which it decreased by the time of chick hatching (0.043 ± 0.006)—this corresponds to the physiological ontogenetic program. In the experimental group, the opposite pattern was observed: expression remained low during embryogenesis (0.067 ± 0.047), but significantly increased by one day of age (0.395 ± 0.181). This observation may indicate that the freezing process does not simply reduce the level of molecular activity, but disrupts the normative ontogenetic regulatory program embedded in the genome. Similar disruptions in ontogenetic patterns of miRNA expression have been previously described under the influence of other stress factors [36], but this is the first time this has been shown in the context of sperm cryopreservation in birds. A parallel increase in miR-301a-5p and a decrease in the level of *TGFB2* gene expression in both generations indicates the preservation of the functional relationship between the microRNA and its target gene. In F0 sperm, *TGFB2* gene expression was reduced by 42% (*p* < 0.05) in the gonads of 10-day F1 embryos—by 29% (*p* = 0.008) in the testes of day-old chicks—by 43% (*p* < 0.0001). This is consistent with the canonical mechanism of miRNA-mediated post-transcriptional repression [13,37]. The *TGFB2* gene encodes the transforming growth factor β-2 protein, which is involved in testicular cell differentiation, proliferation, and apoptosis [14,38]. The TGF-β/Smad signaling pathway is crucial for Sertoli cell function during spermatogenesis [14]. Reduced *TGFB2* expression mediated by miR-301a-5p upregulation after cryopreservation induces cell apoptosis and inhibits proliferation through activation of cyclin-dependent kinase inhibitors (p21WA F1/CIP1, p15INK4b, p27KIP1a) [38].

In contrast to the miR-301a-5p/*TGFB2* pair, the miR-6701-3p/*DMRT1* association exhibits a paradoxical pattern: a 68% reduction in miRNA expression in F1 should result in gene derepression, but a simultaneous 48% reduction in *DMRT1* is observed in embryos (*p* = 0.018). We hypothesize that this may be due to epigenetic downregulation of the *DMRT1* promoter (e.g., via DNA methylation). The *DMRT1* gene is a Z-linked regulator of male sexual differentiation in birds [11,12,39]. Its downregulation even at early stages of development may have long-term consequences: impaired spermatogonial differentiation, delayed meiosis, and decreased fertility in adulthood. It is known that miR-6701-3p microRNA regulates the *DMRT1* gene through a complementary site in the 3′-UTR (position 1456-1463); however, in our study, a double disruption (a decrease in both miRNA and gene) is presumably associated with hypermethylation of the *DMRT1* promoter under the influence of oxidative stress during cryopreservation [39,40,41]. Another interesting observation is the similarity of the dynamics of the *TGFB2* gene and miR-6701-3p under the influence of cryopreservation—both molecules demonstrate a stable decrease in the experimental group at all studied stages both in paternal sperm and in the gonadal tissues of the offspring at the embryonic and postnatal stages (F0: −42% and −59%; F1 embryos: −29% and −93%; F1 chickens: −43% and −68%). This may indicate a coordinated response to cryostress mediated by oxidative damage of common regulatory pathways. Reactive oxygen species (ROS) generated during the freeze–thaw cycle of semen are known to disrupt the function of enzymes involved in miRNA biogenesis, including Drosha and Dicer [28,42]. In addition, ROS damage mitochondrial complexes and plasma membranes of spermatozoa by destroying their lipid composition [1,27,43], which affects the integrity and functionality of germ cells. A parallel decrease in *TGFB2* may be associated with damage to the TGF-β/SMAD signaling pathway, which is sensitive to oxidative stress [44,45].

The identified molecular abnormalities were reflected in the histological structure of the offspring’s gonads. Analysis of gonadal tissue from 10-day-old embryos revealed early morphological abnormalities in the offspring obtained from cryopreserved semen. In the control group, the gonads exhibited a structure typical for this stage of ontogenesis: a symmetrical arrangement of medullary cords, densely packed gonads, pre-Sertoli cells, and seminiferous tubule rudiments. In the experimental group, disorganization of the medullary cords was observed—an uneven distribution of cell clusters, an increase in intertissue space, and a decrease in gonadocyte density. These changes indicate a delay in germ cell differentiation and a disruption in the rate of seminiferous tubule formation at the embryonic stage of development, which is consistent with data on the effects of endocrine disruptors on embryogenesis [46].

By 1 day of age, the differences between the groups became more pronounced. In the testes of the chickens from the experimental group, a significantly lower number of seminiferous tubules was observed in the cross section (4.7 pcs. versus 7.3 pcs.; *p* < 0.05), while their diameter was larger (52.3 µm versus 42.7 µm; *p* < 0.05). The number of gonocytes in the seminiferous tubule exceeded the control by 53% (67.5 pcs. versus 44.2 pcs.; *p* < 0.001). An increase in the diameter of the tubules with a simultaneous decrease in their number may indicate compensatory hypertrophy in response to a decrease in the total number of germinal cells. The accumulation of gonocytes may reflect a delay in their differentiation into spermatogonia, which is consistent with data on impaired spermatogenesis in epigenetic disorders [20,47]. These histological changes correlate with a decrease in *DMRT1* expression at the embryonic stage, since this gene regulates the differentiation of spermatogonia and the formation of seminiferous tubules [11]. Thus, cryopreservation leads to a redistribution of germ cells: a smaller number of tubules is compensated by their increased diameter and an increased local density of gonocytes; thus, the number of gonocytes per unit area of the spermatogenic epithelium of the seminiferous tubule in the control is 0.057 pcs/μm^2^ and 0.064 pcs/μm^2^ in the experiment.

Our data demonstrate that the epigenetic effects of cryopreservation manifest themselves differently at different stages of chick ontogeny. For the *DMRT1* gene, the most pronounced differences between groups were observed at the embryonic stage (a 48% decrease, *p* = 0.018), whereas by day of age, the differences smoothed out and became statistically insignificant (a 12% decrease, *p* = 0.189). This indicates that cryostress primarily disrupts the establishment of the male phenotype, rather than its maintenance in the postnatal period. Similar stage specificity has previously been described for other epigenetic developmental disorders [46]. The embryonic stage represents a critical susceptibility point, when epigenetic modifications can have long-term consequences for the adult phenotype, but this issue requires further testing during puberty.

The mechanisms of transmission of epigenetic consequences from F0 parents to the F1 generation likely involve several interrelated pathways. First, changes in the microRNA profile in F0 sperm, which are introduced into the zygote and regulate early gene expression. Second, disruption of DNA methylation patterns in the promoter regions of the *DMRT1* and *TGFB2* genes under the influence of oxidative stress during cryopreservation. Third, changes in chromatin packaging (histone/protamine ratio) affecting the accessibility of DNA for transcription [7,48]. Avian sperm, as described previously, are characterized by a unique chromatin structure with a high degree of condensation, which may contribute to the maintenance of stable gene expression after a freeze–thaw cycle (Pavlov et al., 1970 [25]). However, our data show that even with such protection, epigenetic disturbances are still transmitted to offspring. The persistence of molecular changes into the postnatal period indicates a potential impact on the phenotypic characteristics of the offspring. In addition, a decrease in *DMRT1* at the embryonic stage may lead to impaired spermatogonial differentiation and reduced fertility in adulthood [47]. In turn, an increase in miR-301a-5p and suppression of the *TGFB2* gene may affect the proliferation and differentiation of germ cells [13]. High interindividual variability in miR-301a-5p expression in the experimental group (CV = 129%) reflects the heterogeneity of the response to cryostress at the molecular level.

## 5. Conclusions

These data provide important evidence of transgenerational effects in birds and contribute to the search for solutions to problems associated with maintaining sperm quality after cryopreservation in poultry farming. Cryopreservation of rooster sperm causes dual disruption of microRNA regulation: direct (miR-301a-5p → *TGFB2*) through classical repression and epigenetic (miR-6701-3p → *DMRT1*) through promoter methylation dominance. The most compelling evidence of a transgenerational effect is the opposite dynamics of miR-301a-5p expression during the transition from embryos to day-old chicks: in the naive group, expression increases 2.98-fold (physiological activation), while in the experimental group, it decreases 5.88-fold (paradoxical suppression). This indicates that cryopreservation not only reduces the level of molecular activity but also disrupts the ontogenetic regulatory program embedded in the genome. These findings call for further study of the long-term phenotypic consequences for offspring and the development of methods to minimize the epigenetic risks of cryopreservation in poultry farming.

## Figures and Tables

**Table 1:** Nucleotide sequence of TaqMan primers and probe used for real-time RT-PCR.

Gene/microRNA Name	Oligonucleotide Sequence (5′-3′)
*TGFB2*	F: GAAGCTTCTGCCTCTCCGTG
	RV: GTCACGCTGTTTCTGGGGTA
*DMRT1*	F: CACACAGATACTGGCCTCGG
	RV: TAAGTCGAGGCACTCAACGC
*GAPDH*	F: CGCCATCACTATCTTCCAGG
	RV: CCTCTGTCATCTCTCCACAGC
gga-miR-301a-5p	SL: GTTGGCTCTGGTGCAGGGTCCGAGGTATTCGCACCAGAGCCAAC AGTAGT
	F: GTGGGTCTGACAATGTTGC
gga-miR-6701-3p	SL: GTTGGCTCTGGTGCAGGGTCCGAGGTATTCGCACCAGAGCCAAC GCGATC
	F: GTGGGGGATTATTTTACAGACA
UPL	F: GTGGGTCTGACAATGTTGC
UPL (TaqMan)	[FAM]TGGCTCTGGTGCGAATAC[BHQ1]
gga-miR-6701-3p mimic	AUUAUUUUACAGACAGAUCGC
gga-miR-301a-5p mimic	UCUGACAAUGUUGCACUACU
U6 (RNU6B)	F: CTCGCTTCGGCAGCACA
	R: AACGCTTCACGAATTTGCGT

**Table 2:** Quality indicators for native and frozen–thawed semen of roosters (F0) (n = 8) (mean ± SE).

Indicator	Native Semen	Frozen–Thawed Semen	*p*-Value
Total motility (TM), %	86.4 ± 2.1	52.7 ± 4.3	<0.001
Progressive motility (PM), %	78.9 ± 2.5	34.2 ± 3.8	<0.001
Viability, %	91.7 ± 1.2	68.3 ± 3.5	<0.001

**Table 3:** Cytometric indices of native and frozen–thawed semen of F0 roosters (n = 8) (mean ± SE).

Indicator	Native Semen	Frozen–Thawed Semen	*p*-Value
Viable cells (Annexin V^−^/PI^−^), %	78.38 ± 1.16	48.35 ± 2.02	<0.001
Apoptosis (Annexin V^+^/PI^−^), %	1.55 ± 0.21	4.19 ± 0.20	<0.001
Mitochondrial membrane potential, (TMRE^+^), %	76.52 ± 0.76	53.26 ± 1.80	<0.001

**Table 4:** Results of histomorphometric evaluation of gonads of 10-day-old embryos obtained from native (control group, n = 15) and frozen–thawed (experimental group, n = 10) semen (F1) (mean ± SE).

Group	Gonad Length, µm	Gonad Width, µm	Number of Gonocytes *, pcs.
F1-control	1360.9 ^a^ ± 19.7	345.4 ^c^ ± 1.0	90.1 ^d^ ± 3.5
CV, %	2.0	0.4	19.7
F1-experimental	1595.9 ^b^ ± 53.4	357.9 ^c^ ± 19.0	93.7 ^d^ ± 2.4
CV, %	18.8	11.9	9.7

Note: * in the field of view at ×1000 magnification (oil) on equal areas (5000 µm^2^). ^ab^ *p* < 0.001; ^c^ and ^d^—no significant.

**Table 5:** Results of evaluation of histological preparations of testes of 1-day-old chicks obtained from native (n = 20) and frozen–thawed (n = 17) semen (F1) (mean ± SE).

Group	Testicle Length, µm	Testicle Width, µm	Number of Seminiferous Tubules in Cross-Section *, pcs.	Diameter of Seminiferous Tubules in Cross-Section, µm	Height of Spermatogenic Epithelium of Testis, µm	Number of Spermatogonia in Cross-Section of Seminiferous Tubule **, pcs.
F1-control	2385.7 ± 232.4	721.5 ± 55.8	7.3 ± 0.8 ^a^	42.7 ± 2.1 ^a^	6.82 ± 0.53	44.2 ± 2.6 ^c^
CV, %	21.8	17.3	43.0	24.3	35.6	29.8
F1-experiment	3270.0 ± 432.0	711.3 ± 57.9	4.7 ± 0.8 ^b^	52.3 ± 2.8 ^b^	7.52 ± 0.9	67.5 ± 4.2 ^d^
CV, %	18.7	10.3	39.9	16.8	33.4	19.7

Note: * in the field of view at ×200 magnification; ** in the field of view at ×1000 magnification (oil); ^ab^ *p* < 0.05 ^cd^ *p* < 0.001.

## Data Availability

The original contributions presented in this study are included in the article. Further inquiries can be directed to the corresponding author.
